# Immuno-Positron Emission Tomography with Zirconium-89-Labeled Monoclonal Antibodies in Oncology: What Can We Learn from Initial Clinical Trials?

**DOI:** 10.3389/fphar.2016.00131

**Published:** 2016-05-24

**Authors:** Yvonne W. S. Jauw, C. Willemien Menke-van der Houven van Oordt, Otto S. Hoekstra, N. Harry Hendrikse, Danielle J. Vugts, Josée M. Zijlstra, Marc C. Huisman, Guus A. M. S. van Dongen

**Affiliations:** ^1^Department of Hematology, VU University Medical CenterAmsterdam, Netherlands; ^2^Department of Medical Oncology, VU University Medical CenterAmsterdam, Netherlands; ^3^Department of Radiology and Nuclear Medicine, VU University Medical CenterAmsterdam, Netherlands; ^4^Department of Clinical Pharmacology and Pharmacy, VU University Medical CenterAmsterdam, Netherlands

**Keywords:** molecular imaging, positron emission tomography, ^89^zirconium, monoclonal antibodies, imaging biomarker, clinical oncology

## Abstract

Selection of the right drug for the right patient is a promising approach to increase clinical benefit of targeted therapy with monoclonal antibodies (mAbs). Assessment of *in vivo* biodistribution and tumor targeting of mAbs to predict toxicity and efficacy is expected to guide individualized treatment and drug development. Molecular imaging with positron emission tomography (PET) using zirconium-89 (^89^Zr)-labeled monoclonal antibodies also known as ^89^Zr-immuno-PET, visualizes and quantifies uptake of radiolabeled mAbs. This technique provides a potential imaging biomarker to assess target expression, as well as tumor targeting of mAbs. In this review we summarize results from initial clinical trials with ^89^Zr-immuno-PET in oncology and discuss technical aspects of trial design. In clinical trials with ^89^Zr-immuno-PET two requirements should be met for each ^89^Zr-labeled mAb to realize its full potential. One requirement is that the biodistribution of the ^89^Zr-labeled mAb (imaging dose) reflects the biodistribution of the drug during treatment (therapeutic dose). Another requirement is that tumor uptake of ^89^Zr-mAb on PET is primarily driven by specific, antigen-mediated, tumor targeting. Initial trials have contributed toward the development of ^89^Zr-immuno-PET as an imaging biomarker by showing correlation between uptake of ^89^Zr-labeled mAbs on PET and target expression levels in biopsies. These results indicate that ^89^Zr-immuno-PET reflects specific, antigen-mediated binding. ^89^Zr-immuno-PET was shown to predict toxicity of RIT, but thus far results indicating that toxicity of mAbs or mAb-drug conjugate treatment can be predicted are lacking. So far, one study has shown that molecular imaging combined with early response assessment is able to predict response to treatment with the antibody-drug conjugate trastuzumab-emtansine, in patients with human epithelial growth factor-2 (HER2)-positive breast cancer. Future studies would benefit from a standardized criterion to define positive tumor uptake, possibly supported by quantitative analysis, and validated by linking imaging data with corresponding clinical outcome. Taken together, these results encourage further studies to develop ^89^Zr-immuno-PET as a predictive imaging biomarker to guide individualized treatment, as well as for potential application in drug development.

## Introduction

In recent years, monoclonal antibodies (mAbs) have become widely used for treatment of cancer. Immunotherapy with mAbs aims for specific targeting and therefore less toxicity compared to chemotherapy. Some mAbs have resulted in a significant improvement of survival, for example the anti-CD20 antibody rituximab for B cell lymphoma (Feugier et al., 2005). However, not all patients benefit from mAb treatment. Monotherapy with the anti-epidermal growth factor receptor (EGFR) antibody cetuximab results in clinical benefit for half of the patients with advanced colorectal cancer (without relevant gene mutations; RAS wild type; Peeters et al., 2015).

Improving response rates by quickly selecting the right drug for the right patient is paramount to reducing unnecessary toxicity and costs. In order to obtain clinical benefit from mAb treatment, the target antigen should be expressed in the tumor and the drug is required to reach and bind to the target (tumor targeting). Absence of target expression on normal tissue is important to limit toxicity of treatment.

Next generation mAbs are aiming for increased potency, for example antibody-drug conjugates (ADC's), mAbs capable of inhibiting immune checkpoints and multi-specific mAbs recognizing at least two different targets (Sliwkowski and Mellman, 2013; Evans and Syed, 2014; Reichert, 2016). The number of novel targeted treatment options increases, however drug development requires time, efforts and significant resources. In addition, investigational drugs are evaluated in large patient cohorts before successful introduction in routine clinical care.

Molecular imaging with ^89^Zr-labeled mAbs, also known as ^89^Zr-immuno-PET, provides a potential imaging biomarker to evaluate tumor targeting of mAbs. This technique is non-invasive and provides whole body information on both target expression and tumor targeting, as opposed to immunohistochemistry on a single biopsy, which only provides information on target expression. Prediction of efficacy and toxicity of mAb treatment by molecular imaging may be used to select individual patients or patient groups for a treatment, or to select promising candidate drugs and their dosing schedule (Ciprotti et al., 2015).

^89^Zr-immuno-PET allows visualization and quantification of biodistribution and tumor uptake. ^89^Zr is considered a suitable radioisotope for this purpose, due to its relatively long half-life (*t* = 78.4 h), which corresponds with the time a mAb needs to reach the target. The use of ^89^Zr as a radiolabel and the coupling of ^89^Zr to mAbs, under Good Manufacturing Practice conditions, have been described previously (Verel et al., 2003; Perk et al., 2010; Vosjan et al., 2010). Harmonization of quantitative ^89^Zr-immuno-PET imaging has also been reported, allowing for broad scale application, e.g., in a multi-center setting (Makris et al., 2014).

Before starting clinical ^89^Zr-immuno-PET trials, the following conditions are essential to allow appropriate interpretation of data. Prerequisites are that the radioimmunoconjugate of interest is stable and has the same binding and biodistribution characteristics as the unlabeled parental mAb. Imaging procedures should be standardized and validated in order to provide reliable quantification. Assuming these requirements are fulfilled, biodistribution and tumor uptake of a ^89^Zr-mAb, defined on PET, can be used as an imaging biomarker for tumor targeting of the “cold” therapeutic antibody. These basic technical aspects of ^89^Zr-immuno-PET have been extensively discussed in a recent review by van Dongen et al. (2015).

Until now, at least 15 clinical ^89^Zr-immuno-PET trials have been reported, see Table 1, providing information on the clinical performance of ^89^Zr-immuno-PET. Therefore, evaluation of the potential and current limitations of this imaging technique seems timely to enable optimal design of future trials. This review summarizes the results from initial clinical ^89^Zr-immuno-PET in oncology, and technical aspects of trial design are discussed.

**Table 1:** **Summary of clinical studies on ^89^Zr-immuno-PET in oncology**.

**Author**	**Year**	**Target**	**mAb**	**Tumor type**	**N**
Börjesson	2006	CD44v6	cmAb U36	Head and neck cancer	20
	2009				
Dijkers	2010	HER2	trastuzumab	Breast cancer	14
Rizvi	2012	CD20	ibritumomab-tiuxetan	B-cell lymphoma	7
Gaykema	2013	VEGF-A	bevacizumab	Breast cancer	23
van Zanten	2013	VEGF-A	bevacizumab	Glioma	3
van Asselt	2014	VEGF-A	bevacizumab	Neuroendocrine tumors	14
Bahce	2014	VEGF-A	bevacizumab	Non-small cell lung cancer	7
Pandit-Taskar	2014	PSMA	Hu-J591	Prostate cancer	50
	2015				
Den Hollander	2015	TGF-β	fresolimumab	Glioma	12
Gaykema	2015	HER2	trastuzumab	Breast cancer	10
		VEGF-A	bevacizumab		6
Gebhart	2015	HER2	trastuzumab	Breast cancer	56
Lamberts	2015	MSLN	MMOT0530A	Pancreatic cancer	11
				Ovarian cancer	4
Menke-van der Houven van Oordt	2015	EGFR	cetuximab	Colorectal cancer	10
Muylle	2015	CD20	rituximab	B-cell lymphoma	5
Oosting	2015	VEGF-A	bevacizumab	Renal cell carcinoma	22

## ^89^Zr-labeled anti-CD44v6 mAb in head and neck cancer

^89^Zr-immuno-PET is considered to be an attractive imaging technique for whole body tumor detection, due to the combined sensitivity of PET and the specificity of the mAb. Assessment of the mAb biodistribution to confirm specificity is particularly of interest to qualify the suitability of the mAb for therapy.

Börjesson et al. reported on the first clinical ^89^Zr-immuno-PET study ever (Börjesson et al., 2006). In this study, twenty pre-operative patients with head and neck squamous cell carcinoma (HNSCC) were included. Immuno-PET with ^89^Zr-labeled chimeric mAb U36 (cmAb U36) was investigated in order to improve tumor detection of HNSCC, especially in lymph nodes, and to assess the targeting potential of the mAb for therapy. cmAb U36 targets the v6 region of CD44 (cluster of differentiation; CD44v6). Homogeneous expression of CD44v6 has been observed in several malignancies, including HNSCC, lung, skin, esophagus, and cervix carcinoma. Expression of CD44v6 has also been reported in normal epithelial tissues, such as skin, breast, and prostate myoepithelium, and bronchial epithelium.

Administration of 74 megabecquerel (MBq) ^89^Zr-mAb U36 (10 mg) appeared to be safe, as no adverse reactions were observed. A human anti-chimeric antibody response was reported in 2 patients, which was not directed to the chelate, but to the protein part of the conjugate. ^89^Zr-immuno-PET scans were visually scored. All primary tumors were detected and ^89^Zr-immuno-PET performed equally to computed tomography (CT) and magnetic resonance imaging (MRI) for detection of lymph node metastasis. Although, biopsies were obtained in this study to confirm tumor localization, immunohistochemistry for CD44v6 was not performed, as in literature >96% of HNSCC show CD44v6 expression by at least 50% of the cells. This study shows that immuno-PET with ^89^Zr-cmAb U36 can be used as an imaging modality for tumor detection. However, traditional imaging techniques as ^18^F-fluoro-deoxy-glucose(FDG)-PET (Mak et al., 2011) and sentinel node procedures for assessment of lymph node status remain standard of care for tumor detection in HNSCC patients, as the added value of immuno-PET with ^89^Zr-cmAb U36 has not been demonstrated.

In a separate publication, biodistribution, radiation dose and quantification of ^89^Zr-labeled cmAb U36 were reported for the same patient cohort (Börjesson et al., 2009). ^89^Zr-cmAb U36 in blood pool, lungs, liver, kidneys and spleen decreased over time. Increasing uptake of ^89^Zr-cmAb U36 over time was seen only at tumor sites and in the thyroid of some patients (suggesting expression of CD44v6 in thyroid of these patients). Although expression of CD44v6 in normal epithelial tissue has been described, no obvious targeting of ^89^Zr-mAb U36 was observed in the skin. However, Tijink et al. reported a fatal adverse event due to skin toxicity after treatment with the ADC bivatuzumab mertansine, a humanized anti-CD44v6 mAb conjugated to a potent maytansine derivate (Tijink et al., 2006). This toxicity profile, most probably due to expression of CD44v6 in the skin, was not predicted based on assessment of biodistribution of ^89^Zr-cmAbU36 as reported by Börjesson et al. (2009). Among others, this may be due to detection limitations of PET, inter-individual differences in target expression, or differences in biodistribution between the mAb and the ADC.

A technical aspect of this first ^89^Zr-immuno-PET study to be considered is the rationale for the total protein dose of cmAb U36 administered, since tumor uptake, tumor to non-tumor ratio's, as well as tumor visualization might be protein dose dependent. This protein dose of 10 mg was chosen as previous studies observed that biodistribution was not mAb-dose dependent within the range of 2–52 mg (de Bree et al., 1995; Colnot et al., 2000; Stroomer et al., 2000).

Finally, good agreement was reported for image-derived quantification of blood pool radioactivity as well as tumor uptake of ^89^Zr-cmAb U36 compared to *ex vivo* radioactivity measurements in, respectively, venous blood samples and biopsies from surgical tumor resection. This is an important achievement in performance, showing accurate quantification of ^89^Zr-mAb with PET.

## ^89^Zr-labeled trastuzumab in breast cancer

Treatment with trastuzumab, which targets the human epidermal growth factor receptor 2 (HER2), has improved the prognosis for patients with HER2-positive breast cancer (Moja et al., 2012) and gastric cancer (Gong et al., 2016). HER2 is involved in cell survival, proliferation, cell maturation, metastasis, angiogenesis and has anti-apoptotic effects. It is also expressed in other malignancies, including ovarian and endometrial carcinoma, and in normal epithelial cells and hematopoietic cells (Leone et al., 2003). It is known that the extracellular domain of HER2 can enter the circulation after shedding from the surface of tumor cells (Tse et al., 2012).

Currently assessment of HER2 status is performed with immunohistochemistry (IHC) or fluorescent in situ hybridization on tumor biopsies. Some studies have shown up to 15% intra-individual heterogeneity in HER2 status between primary tumors and metastases (Lindstrom et al., 2012) leading to the recommendation to repeat biopsies to assess HER2 status during the course of the disease. As some tumor lesions are inaccessible for biopsies and it is impossible to biopsy every tumor lesion to assess heterogeneity, there is a need for a non-invasive technique to assess whole body HER2 status for diagnosis, staging and to guide individualized treatment.

### ^89^Zr-trastuzumab-PET for whole body assessment of HER2 target status

Dijkers et al. reported a feasibility study to determine optimal dosage and time of administration of ^89^Zr-trastuzumab (37 MBq) to enable PET visualization and quantification of tumor lesions in 14 patients with HER2-positive metastatic breast cancer (Dijkers et al., 2010).

**Figure 1:**
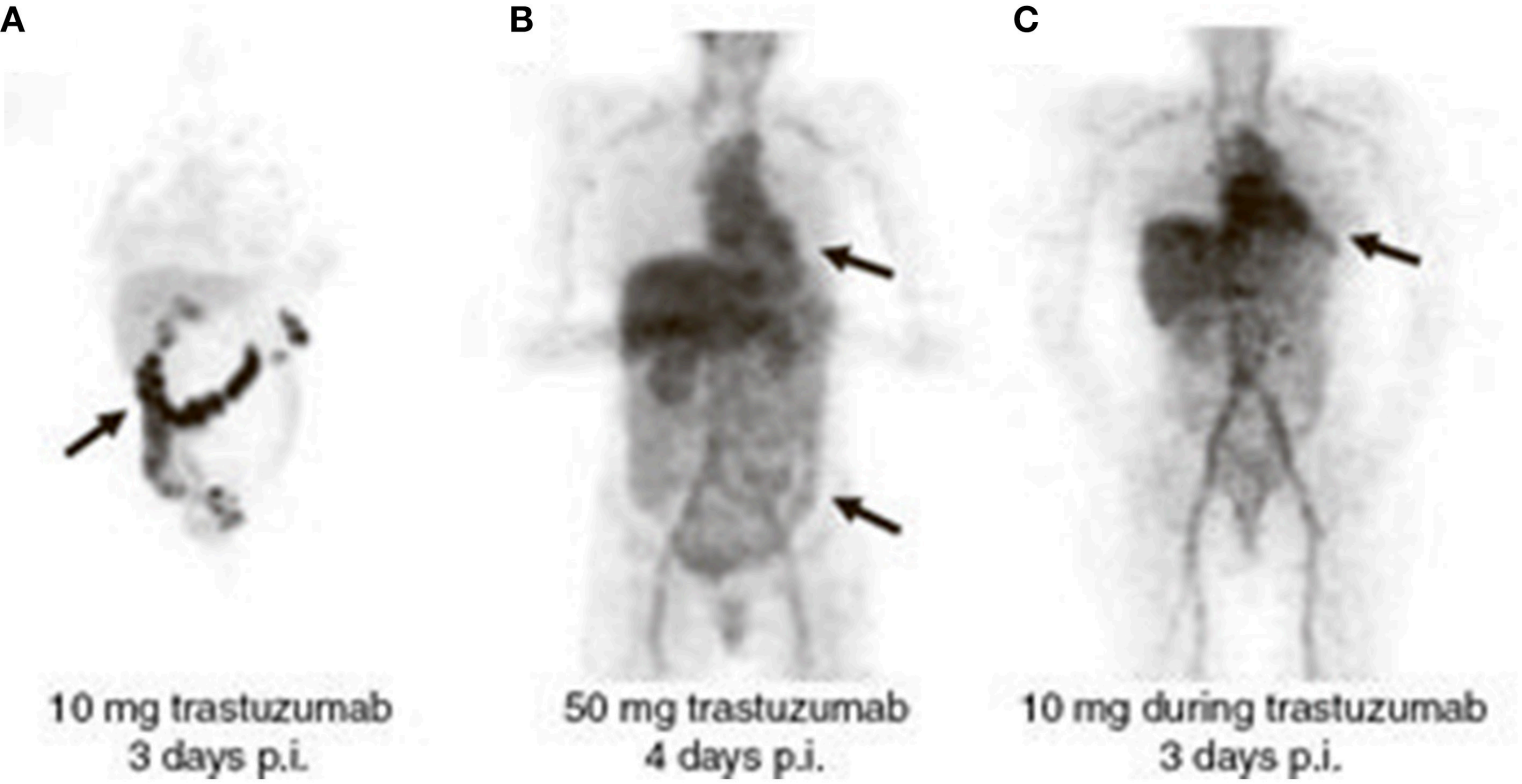
**Dose-dependent biodistribution and clearance of ^89^Zr-trastuzumab**. Radioactivity in the blood pool and intestinal excretion are indicated by arrows. **(A)** Trastuzumab-naïve patient, imaging dose = 10 mg. **(B)** Trastuzumab-naïve patient, imaging dose = 50 mg. **(C)** Patient on trastuzumab treatment, imaging dose = 10 mg. Reprinted with permission from Dijkers et al. (2010).

Trastuzumab naïve patients who received -^89^Zr-trastuzumab (10 mg; *n* = 2), showed relatively high liver uptake and pronounced intestinal excretion, with low blood pool activity, indicating rapid clearance. This rapid clearance was most probably due to complex formation of trastuzumab with extracellular HER2 domains shed in the plasma. For optimal imaging, trastuzumab naïve patients required a total dose of 50 mg trastuzumab (*n* = 5). This resulted in less liver uptake, lower intestinal excretion and increased blood pool activity, as illustrated by Figures 1A,B. This dose was considered the optimal dose, as good tumor-to-non-tumor ratio and favorable biodistribution were observed, although higher doses were not evaluated due to expected target saturation. Patients already on trastuzumab treatment received a dose of 10 mg trastuzumab. As these patients (*n* = 7) showed minimal intestinal excretion and slow blood clearance, this was considered an adequate dose, see Figure 1C. This study illustrates a dose-dependent relationship between imaging dose and biodistribution of ^89^Zr-trastuzumab.

Best timing for evaluation of tumor uptake of ^89^Zr-trastuzumab was 4–5 days post injection (p.i.) while scans performed at day 6 or 7 p.i. yielded decreased image quality because of insufficient counting statistics.

^89^Zr-trastuzumab-PET allowed detection of most of the known lesions and some that had remained unnoticed with CT, MRI or bone scans. In 6 of the 12 patients not all known lesions were detected. Liver lesions were missed in 3 out of 7 patients, possibly due to the high background activity in normal liver tissue, which is involved in mAb catabolism. Interestingly, while poor penetration of trastuzumab in the brain was expected, in 3 patients brain lesions could be visualized. This might be due to disruption of blood-brain barrier in brain metastasis. A limitation of this study was the lack of biopsies for confirmation of the HER2 status of immuno-PET negative lesions.

Tumor lesions showing no uptake of ^89^Zr-trastuzumab may be due to suboptimal imaging conditions, as illustrated by a case report of a trastuzumab naïve breast cancer patient (Oude Munnink et al., 2010). Thirty seven MBq trastuzumab (50 mg) was administered and a PET scan was obtained 2 days p.i. ^89^Zr activity concentration in bloodpool was low, whereas massive uptake was observed in liver metastases (48% of the injected dose) as well as intestinal uptake, suggesting intestinal excretion. Known bone metastases were hardly visible. This might be the result of an extensive tumor load and/or soluble HER2, which reduces uptake in other tumor lesions, due to an insufficient amount of trastuzumab. After administration of 220 mg unlabeled trastuzumab, immediately followed by 10 mg ^89^Zr-trastuzumab, liver uptake was lowered (33% of the injected dose) and an increase in uptake in the other tumor lesions, such as bone metastases, was observed. Based on a theoretical calculation the authors conclude that a dose of 280 mg trastuzumab could only saturate 47% of all HER2 present in the liver metastases of this patient, indicating a higher dose of trastuzumab is required to saturate lesions in case of extensive HER2-positive tumor load. Based on this important observation that pharmacokinetics and organ distribution can be influenced by the extent of tumor load, dosing of trastuzumab for metastastic breast cancer should be reconsidered. An individualized dosing schedule of trastuzumab based on tumor load, guided by ^89^Zr-trastuzumab-PET, instead of patient weight, might improve efficacy of treatment.

### ^89^Zr-trastuzumab to assess response by alteration of antigen expression

Gaykema et al. evaluated ^89^Zr-trastuzumab-PET to determine alteration of HER2 expression after anti-angiogenic treatment with the novel heat shock protein 90 (HSP90) inhibitor NVP-AUY922 in 10 patients with HER2-positive breast cancer (Gaykema et al., 2014). HSP90 inhibition can deplete client proteins like HER2. This study was performed with 37 MBq ^89^Zr-trastuzumab (50 mg), while NVP-AUY922 was administered i.v. in a weekly schedule of 70 mg/m^2^. Change in tumor uptake of ^89^Zr-trastuzumab at baseline vs. 3 weeks on treatment was correlated to change in size on CT after 8 weeks treatment. This feasibility study suggests that ^89^Zr-immuno-PET can be used to monitor alteration of antigen expression and supports further evaluation of ^89^Zr-trastuzumab-PET in providing insight in treatment response of novel anti-cancer agents like the HSP90 inhibitor NVP-AUY922 in larger studies.

### ^89^Zr-trastuzumab-PET as predictive imaging biomarker for ADC treatment

**Figure 2:**
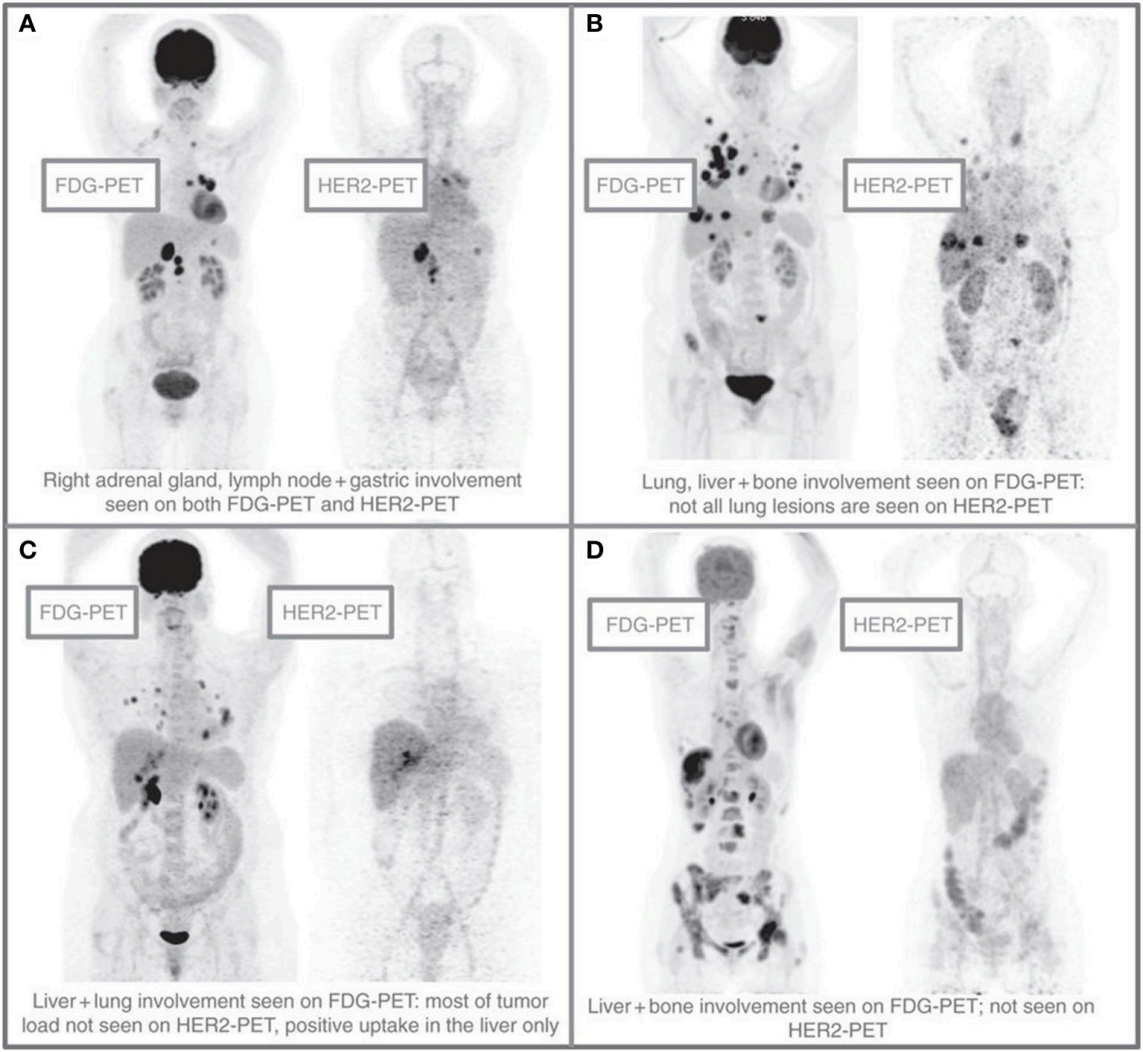
**Patterns of HER2-PET/CT confronted with FDG-PET/CT, maximum intensity projection**. Lesion uptake was considered pertinent when visually higher than blood pool. **(A)** Entire tumor load showed pertinent tracer uptake. **(B)** Dominant part of tumor load showed tracer uptake. **(C)** Minor part of tumor load showed tracer uptake. **(D)** Entire tumor load lacked tracer uptake. Reprinted with permission from Gebhart et al. (2016).

Recently, the ADC trastuzumab-emtansine (T-DM1) was approved for treatment of patients with progression of HER2-positive breast cancer, previously treated with trastuzumab-based therapy. The ZEPHIR study investigated the use of ^89^Zr-trastuzumab-PET, combined with early response assessment with FDG-PET, as a predictive imaging biomarker for treatment with T-DM1 (Gebhart et al., 2016). In this study intra- and interpatient heterogeneity in HER2 mapping of metastatic disease was also explored, see Figure 2. The study was performed by administration of 37 MBq ^89^Zr-trastuzumab (50 mg). ^89^Zr-trastuzumab-PET scans were acquired 4 days p.i. and visually scored. After 1 cycle of T-DM1 an early metabolic response was evaluated by FDG-PET. Clinical outcome after treatment with 3 cycles of T-DM1 was evaluated by CT.

For 55 evaluable patients with HER2-positive metastatic breast cancer, assessment of ^89^Zr-trastuzumab uptake resulted in a positive predictive value of 72% and a negative predictive value of 88% for prediction of clinical outcome. For early metabolic response assessment with FDG-PET the positive predictive value was 96% and the negative predictive value was 83%. Intrapatient heterogeneity in tumor uptake was observed in 46% of patients, as illustrated by Figure 2. When combining ^89^Zr-trastuzumab-PET with early FDG-PET response after 1 cycle of T-DM1, a negative predictive value of 100% was obtained for all concordant patients (with both a negative ^89^Zr-trastuzumab-PET, as well as absence of response on early FDG-PET). This strategy of combining HER2-PET with early FDG-PET response monitoring was able to separate patients with a median time to treatment failure of 2.8 month from patients with a median time to treatment failure of 15 months.

It is not known why 2/16 patients with a negative ^89^Zr-trastuzumab-PET did show response on treatment with T-DM1. Some possibilities are lack of receptor overexpression, receptor masking, or an induced response despite low HER2 expression due to the extreme potency of T-DM1. Another possibility is that DM1 becomes released in the circulation and exerts some antitumor activity. Absence of tumor uptake can also be explained by an insufficient tracer dose due to the extent of tumor load or the amount of soluble HER2 in these patients.

These results support that pre-treatment imaging of HER2-targeting is a promising tool to improve the understanding of tumor heterogeneity in metastatic breast cancer and to select patients who are deemed not to benefit from T-DM1. This might avoid toxicity and costs of T-DM1 and improve patient outcome by switching sooner to a more effective therapy (personalized medicine). A plausible explanation for the added value of early FDG-PET is that although target expression of HER2 is a prerequisite for clinical benefit, even with adequate targeting intracellular resistance mechanisms may be responsible for treatment failure. The authors recommend a future randomized trial with cost-effectiveness as secondary endpoint, to test the concept of interrupting T-DM1 treatment after 1 cycle in case of FDG-PET non-responsiveness, which can be expected in 20% of the patients. As such, this trial paved the road toward improved individualization of anti-HER2 therapy.

## ^89^Zr-labeled bevacizumab in breast cancer, lung cancer, renal cell carcinoma, neuroendocrine tumors, and pontine glioma

Another target for treatment of breast cancer and many other tumor types is vascular endothelial growth factor A (VEGF-A), which is involved in tumor angiogenesis. Overexpression of VEGF-A has been reported in malignant breast tumors and ductal carcinoma in situ and has been related to aggressiveness of the disease. Bevacizumab is a mAb that targets all splice variants of VEGF-A, both small isoforms which can diffuse freely in the circulation, as well as larger isoforms which are primarily matrix-bound. Despite the fact that VEGF-A is not a membrane target like HER2, it is partly associated with the tumor blood vessels and to some extent with the extracellular matrix of tumor cells, which could enable imaging of tumor lesions.

It can be hypothesized that local VEGF-A concentration reflects whether tumor progression is driven by angiogenesis and if anti-angiogenic treatment is likely to be effective. Therefore, ^89^Zr-bevacizumab-PET is of interest for several applications: biological characterization of tumors, prediction of therapeutic outcome, and treatment evaluation of VEGF-A targeting drugs.

### ^89^Zr-labeled bevacizumab in breast cancer

Gaykema et al. performed a study to assess whether VEGF-A can be visualized by ^89^Zr-bevacizumab-PET in patients with primary breast cancer who were scheduled for surgery (Gaykema et al., 2013). In this study 37 MBq ^89^Zr-labeled bevacizumab (5 mg) was administered and PET scans were acquired at 4 days p.i. The same dose was used in a previous study with ^111^Indium-labeled bevacizumab, which visualized all known melanoma lesions (Nagengast et al., 2011). Tumor uptake of ^89^Zr-bevacizumab was observed in 25 of 26 tumors in 23 patients with primary breast cancer. The false-negative tumor was a 10 mm VEGF-A-positive invasive ductal carcinoma. Besides assessment of tumor uptake in the primary tumors, uptake of ^89^Zr-bevacizumab in lymph node metastasis regions was evaluated. 4 of 10 metastasis-involved lymph node regions were detected in 9 patients. Zero of 4 axillary regions with lymph node metastases were detected by ^89^Zr-bevacizumab-PET.

For all available tumors (*n* = 25) VEGF-A expression was quantified by enzyme-linked immunosorbent assay. ^89^Zr-bevacizumab uptake in tumors correlated with the VEGF-A tumor levels measured. Microvessel density on immunohistochemistry was not correlated with ^89^Zr-bevacizumab uptake. This was the first clinical study showing a significant correlation between antigen expression and tumor uptake of the mAb. The fact that VEGF-A is not/hardly expressed in normal tissue, while the antigen might be well accessible for mAbs in tumor tissue, might be favorable factors for finding such a correlation. This observation opens avenues for the use of ^89^Zr-bevacizumab-PET for response monitoring in therapeutic strategies aiming to downregulate VEGF-A.

The effect of the HSP90 inhibitor NVP-AUY922 on alteration of VEGF-A status was evaluated in the same study discussed earlier for ^89^Zr-trastuzumab in breast cancer (Gaykema et al., 2014). However, ^89^Zr-bevacizumab-PET was not able to predict treatment outcome measured by CT in patients with estrogen-receptor-positive breast cancer (*n* = 6). Possible explanations are that most lesions found on ^89^Zr-bevacizumab PET were bone lesions and not measurable on CT, and that HIF1a is likely a less prominent client protein of HSP90 than HER2. Target expression may be dependent on tumor type, as ^89^Zr-bevacizumab uptake in breast tumors was consistently lower than in renal cell cancer.

### ^89^Zr-labeled bevacizumab in lung cancer

Bevacizumab is also used for treatment of non-small cell lung carcinoma and ^89^Zr-bevacizumab is a potential predictive imaging biomarker for this patient group. In a pilot study it was evaluated whether tumor uptake of ^89^Zr-bevacizumab could be visualized and quantified in 7 patients with lung cancer (Bahce et al., 2014). Moreover, in this study the correlation between tumor uptake of ^89^Zr-bevacizumab and response to a bevacizumab-based treatment regimen was explored. ^89^Zr-bevacizumab PET was performed at day 4 and 7 after injection of 37 MBq ^89^Zr-labeled bevacizumab (5 mg), one week prior to start of induction therapy with carboplatin, paclitaxel and bevacizumab (15 mg/kg), followed by bevacizumab maintenance upon non-progression after 4 cycles.

All tumor lesions showed visible ^89^Zr-bevacizumab uptake. Tumor-to-blood ratios increased from 1.2 ± 0.4 to 2.2 ± 1.2 between day 4 and 7 p.i. Tumor lesions had an approximately four times higher ^89^Zr-bevacizumab uptake compared to non-tumor background tissues (muscle, healthy lung and fatty tissue). A positive trend, but no significant correlation, was observed for tumor uptake and progression-free survival and overall survival after combined chemo-immunotherapy.

A limitation of this study design is that no distinction is possible between therapeutic contribution of chemotherapy and immunotherapy, as this is a combined treatment regimen. Therefore, further ^89^Zr-bevacizumab-PET studies are required to assess VEGF-target status after combination treatment, as a response predictor for effectiveness of subsequent bevacizumab maintenance therapy.

As all lesions were visualized with a total protein dose of 5 mg ^89^Zr-bevacizumab, and no targeting of normal tissues became apparent, there is no indication of an antigen sink for this mAb. A limitation of the current study was that no extra biopsies were obtained to confirm VEGF-A tumor expression as driver for ^89^Zr-bevacizumab uptake and therapeutic response.

### ^89^Zr-labeled bevacizumab in renal cell carcinoma

Oosting et al. investigated ^89^Zr-bevacizumab-PET in patients with metastatic renal cell carcinoma as an imaging modality for treatment evaluation of anti-angiogenic drugs (Oosting et al., 2015).

Patients were randomized between treatment with bevacizumab (10 mg/kg intravenously every 14 days) combined with interferon-α (3 million IU, 3 times per week) (*n* = 11) or sunitinib (50 mg orally, daily for 4 weeks followed by 2 weeks off treatment) (*n* = 11), which is a multi-targeted receptor tyrosine kinase inhibitor. At baseline, and 2 and 6 weeks after treatment, PET scans were acquired after administration of 37 MBq ^89^Zr-labeled bevacizumab (5 mg).

Tumor lesions were visualized in all patients (in total 125 lesions), including 35 that had not been detected by CT. 19 lesions were outside the field of view of the CT, including 5 brain lesions in 3 patients (two had known brain metastasis). Remarkable interpatient and intrapatient heterogeneity in tumor uptake of ^89^Zr-bevacizumab was observed. A strong decrease in tumor uptake of −47.0 % at week 2 was observed for patients on bevacizumab/interferon-α treatment, with an additional change of −9.7% at week 6. For patients on sunitinib treatment, a mean change in tumor uptake of −14.3% at week 2 and a rebound of +72.6% at week 6 was reported (after 2 drug-free weeks).

Change in tumor uptake of ^89^Zr-bevacizumab did not correlate with time to progression. Baseline tumor uptake of ^89^Zr-bevacizumab corresponded with longer time to progression. Although, reduced ^89^Zr-bevacizumab tumor uptake after treatment might be caused by saturation due to treatment with unlabeled bevacizumab, other clinical studies have suggested that bevacizumab-induced vascular changes do occur after treatment.

Further studies are required to assess whether baseline tumor uptake of ^89^Zr-bevacizumab can be used to predict benefit from anti-angiogenic treatment. Heterogeneity in tumor uptake of ^89^Zr-bevacizumab may offer a possibility to differentiate patients groups based on tumor biology and to guide the choice between anti-angiogenic and other treatment strategies.

### ^89^Zr-labeled bevacizumab in neuroendocrine tumors

In another feasibility study with ^89^Zr-bevacizumab, the effect of everolimus, a mammalian target of rapamycin inhibitor, on tumor uptake was investigated in patients with advanced progressive neuroendocrine tumors (NET) (van Asselt et al., 2014). As everolimus can reduce VEGF-A production, it was evaluated whether NET lesions can be visualized by ^89^Zr-bevacizumab PET and whether tumor uptake of ^89^Zr-bevacizumab decreases from baseline to week 2 and 12 during everolimus therapy (10 mg orally once daily). This study was also performed with 37 MBq ^89^Zr-labeled bevacizumab (5 mg), with imaging 4 days p.i. In 4 of 14 patients no tumor lesions could be visualized with ^89^Zr-bevacizumab-PET, in the remaining patients only 19% of tumor lesions ≥1 cm (63 lesions in total) known by CT were positive at PET, demonstrating variable ^89^Zr-bevacizumab tumor uptake in NET patients. ^89^Zr-bevacizumab uptake diminished during everolimus treatment with a mean of −7% at 2 weeks and −35% at 12 weeks. Change in tumor uptake correlated with treatment outcome, assessed by CT after 3 months. There was no correlation between baseline tumor uptake and change of tumor size as assessed by CT, indicating that ^89^Zr-bevacizumab-PET was not qualified for response prediction before therapy.

Interestingly, in 4 of 14 patients no visual uptake was observed, while no patient had progressive disease after 3 and 6 months of treatment with everolimus. This might indicate that everolimus exerts also other mechanisms of action than just reduction of VEGF-A, or reflect that NETs are slow growing tumors.

### ^89^Zr-labeled bevacizumab in pontine glioma

For brain tumors like diffuse intrinsic pontine glioma in children, it is not known whether targeted drugs actually can reach the tumor. Nevertheless, several studies are ongoing to investigate treatment with bevacizumab for this indication. The first report ever on molecular imaging in children, was a feasibility study on the therapeutic potential of bevacizumab and toxic risks, due to VEGF-A expression in normal organs in children with pontine glioma (van Zanten et al., 2013). 3 patients, aged 6, 7, and 17 years received 0.9 MBq/kg (range 18–37 MBq) ^89^Zr–bevacizumab (0.1 mg/kg). Whole body PET scans were obtained at 1, 72, and 144 h p.i. Tumor uptake of ^89^Zr-bevacizumab was observed in 2 of 3 patients, limited to the T1-MRI contrast-enhanced part of the tumor. These findings suggest that disruption of the blood-brain barrier, as indicated by MRI contrast, is necessary for effective tumor targeting by ^89^Zr-bevacizumab. Uptake in normal organs was highest in the liver, followed by the kidneys, lungs, and bone marrow. This study illustrates that also in children ^89^Zr-immuno-PET is a feasible procedure, and has potential as a response and toxicity predictor for treatment with bevacizumab and other targeted drugs.

## ^89^Zr-labeled fresolimumab in high-grade glioma

As indicated before, mAbs might be prevented from reaching tumor lesions in the brain by impermeability of the blood-brain barrier, while tumor targeting is a prerequisite for effective treatment. An appealing target molecule for treatment of high-grade glioma is transforming growth factor β (TGF-β), which functions as a tumor promotor and induces proliferation and metastasis, while suppressing the immune response. TGF-β and its receptors are overexpressed in high-grade glioma and can be targeted with several types of TGF-β inhibitors. Fresolimumab is a mAb capable of neutralizing all mammalian isoforms of TGF-β (i.e., 1, 2, and 3) and has been investigated in phase I trials with patients with melanoma, renal cell carcinoma and in a phase II trial in patients with mesothelioma.

Den Hollander et al. investigated uptake of ^89^Zr-fresolimumab in 12 patients with recurrent high-grade glioma and assessed clinical outcome after fresolimumab treatment (den Hollander et al., 2015). In this study an imaging dose of 37 MBq ^89^Zr-fresolimumab (5 mg) was used before start of treatment (5 mg/kg i.v. every 3 weeks) and PET scans were obtained for all patients on day 4 p.i., while 4 patients also received a scan at day 2 p.i.

In all patients uptake of ^89^Zr-fresolimumab was observed in brain tumor lesions (*n* = 16), while in 8 patients not all known brain tumor lesions were visualized with ^89^Zr-fresolimumab-PET (mostly small lesions, < 10 mm on MRI). The three lesions larger than 10 mm that were missed by ^89^Zr-fresolimumab-PET were suspected to represent radionecrosis instead of viable tumor tissue (therefore probably lacking TGF-β expression), based on previous irradiation or disappearance on follow-up MRI. Tumor-to-blood ratios increased from day 2 to 4 p.i. in patients who underwent whole body PET scans (*n* = 4), which was considered to be suggestive for tumor-specific TGF-β-driven mAb uptake. All patients showed progressive disease on fresolimumab treatment, therefore no correlation between tumor uptake of ^89^Zr-fresolimumab and clinical response was observed. Because of absence of clinical benefit the study was closed after the first 12 patients.

In conclusion, this study showed that ^89^Zr-fresolimumab reaches brain tumor lesions. mAb treatment with TGF-β targeting drugs remains an interesting approach for treatment of high-grade glioma, especially since targeting of brain tumor lesions has been observed by ^89^Zr-fresolimumab-PET. Increase in tumor to blood ratios suggests specific tumor uptake, although non-specific antibody uptake due to disruption of the blood-brain barrier cannot be excluded.

## ^89^Zr-labeled cetuximab in colorectal carcinoma

Another target antigen of interest is EGFR, which can be targeted with cetuximab. Binding of cetuximab to EGFR prevents growth factor binding to the receptor, induces receptor internalization, and causes inhibition of the receptor tyrosine kinase activity. In this way cetuximab interferes with cell growth, differentiation and proliferation, apoptosis and cellular invasiveness. Colorectal cancer with RAS wild type can be effectively treated with cetuximab, while it is known that patients with a K-RAS or N-RAS mutation do not respond to anti-EGFR treatment (van Cutsem et al., 2011). Only patients with RAS wild type colorectal cancer are eligible for anti-EGFR treatment. However, even in this selected patient group efficacy of single agent cetuximab remains limited, as clinical benefit is observed in only half of the patients (Peeters et al., 2015). Additional growth activating mutations or insufficient tumor targeting may affect clinical efficacy. As EGFR is highly expressed on hepatocytes in normal liver tissue, this might lead to sequestration of anti-EGFR-mAbs shortly after administration and interfere with effective tumor targeting.

Assuming that response to treatment is dependent on uptake of cetuximab in tumor lesions, only patients in whom tumor targeting can be confirmed will be susceptible to treatment. Menke-van der Houven van Oordt et al. performed a feasibility study in 10 patients with advanced colorectal cancer to investigate biodistribution and tumor uptake of ^89^Zr-cetuximab and evaluated ^89^Zr-cetuximab as a predictive imaging biomarker (Menke-van der Houven van Oordt et al., 2015). While blood pool, spleen, kidney and lung activity decreased, uptake in the liver increased during the first 2 days, after which a plateau was reached. Total radioactivity derived from the whole body PET images decreased due to gastro-intestinal excretion, while no excretion via the bladder was observed.

Tumor uptake was visible in 6 of 10 patients, of which 4 had clinical benefit. In a patient with 2 lung lesions, one lesion could be visualized, while the other could not, possibly indicating intra-individual heterogeneity of receptor expression or an effect of tumor size (the lesion not visualized was smaller). Of the remaining 4 out of 10 patients without tumor uptake, 3 had progressive disease and 1 had clinical benefit without visible ^89^Zr-cetuximab uptake. Possibly, the amount of cetuximab that reached the latter tumor was insufficient for visual detection, but did induce anti-tumor activity. This example indicates that for appropriate interpretation of tumor uptake-response relationships it is of paramount importance that ^89^Zr-cetuximab and unlabeled cetuximab show exactly the same biodistribution.

Altogether these results support further investigations in a larger cohort to assess whether ^89^Zr-cetuximab can discriminate between cetuximab responding and non-responding patients. Currently a follow-up study, including dose escalation for patients without visible tumor uptake of ^89^Zr-cetuximab and assessment of cetuximab concentrations in tumor biopsies, is ongoing (ClinicalTrials.gov Identifier: NCT02117466). A limitation of ^89^Zr-cetuximab-PET reported by the authors is its inability to detect tumor lesions in the liver. In contrast to ^89^Zr-trastuzumab, ^89^Zr-cetuximab specifically accumulates in normal liver tissue resulting in spillover when quantifying hepatic lesions. Important technical aspects to consider are whether the imaging dose (^89^Zr-labeled cetuximab) and the therapeutic dose (unlabeled cetuximab) show similar biodistribution, and if the degree of similarity can be influenced by the sequence of administration.

In this study patients were treated with a cold therapeutic dose of cetuximab (500 mg/m^2^), within 2 h followed by the infusion of 37 MBq ^89^Zr-cetuximab (10 mg). It was assumed that within this time frame the therapeutic dose and the imaging dose behave as if injected simultaneously due to slow pharmacokinetics. Sequential administration was chosen to make radiation safety precautions during administration of ^89^Zr-cetuximab easier.

Previous studies with ^111^In-cetuximab (C225) in patients with squamous cell lung carcinoma have indicated a dose-dependent biodistribution, showing liver sequestration of ^111^In-cetuximab, which decreased with increasing doses of unlabeled cetuximab (up to 300 mg), while tumor uptake increased (Divgi et al., 1991). To get better insight in the dose-dependent biodistribution of ^89^Zr-cetuximab, Menke-van der Houven van Oordt et al. administered a scouting dose of 0.1 mg ^89^Zr-cetuximab before the dose of unlabeled cetuximab in 3 patients (Menke-van der Houven van Oordt et al., 2015). Blood samples 2 and 3 h post injection of the scouting dose revealed that only < 10% of the injected dose of ^89^Zr-cetuximab was left in the blood circulation. When subsequently cold cetuximab (500 mg/m^2^) was administered, ^89^Zr-cetuximab reappeared in the blood, indicating that it can be reversibly extracted, most probably by the liver. On the other hand, the biological half-life of ^89^Zr-cetuximab, if administered directly after the unlabeled dose, was comparable with the half-life as reported for unlabeled cetuximab. This indicates that upon such sequential administration ^89^Zr-cetuximab indeed reflects the biodistribution of unlabeled cetuximab. Future studies are required to assess to which extent sequential administration of imaging and therapeutic doses influences tumor biodistribution and tumor uptake of ^89^Zr-cetuximab.

## ^89^Zr-labeled anti-PSMA in prostate cancer

Current clinical challenges in imaging of metastatic prostate cancer include limited sensitivity and specificity to detect early metastases (especially in bone) and active disease and to monitor treatment of metastatic prostate cancer. The humanized mAb huJ591 targets the extracellular domain of prostate-specific membrane antigen (PSMA), a transmembrane glycoprotein expressed by both benign and malignant prostate epithelial cells. Nearly all prostate cancers express PSMA. Upon binding, the huJ591-PSMA complex becomes rapidly internalized. Binding of anti-PSMA mAbs to non-prostate tissue, as the liver, duodenal epithelial (brush border) cells and proximal tubule cells in the kidney, has been observed, as well as binding to tumor-associated neovasculature in other solid malignancies, including clear cell renal carcinoma, colon and breast carcinoma (Chang, 2004).

Recently, Pandit-Taskar et al. performed a clinical study with ^89^Zr-labeled huJ591 in 50 patients with castrate-resistant metastatic prostate cancer (Pandit-Taskar et al., 2015). Results of the first 10 patients were reported separately, including assessment of optimal imaging time post-injection for lesion detection of ^89^Zr-huJ591 PET imaging (Pandit-Taskar et al., 2014). These 10 patients received 4 scans within 8 days after injection. Based on optimal tumor-to-background ratios, the other 40 patients were imaged once at 6–8 days p.i. In this ^89^Zr-immuno-PET study a total mAb dose of 25 mg huJ591 was used, of which 1.7 mg was ^89^Zr-labeled (203 MBq). This dose was based on prior studies with ^111^In-J591 and ^177^Lu-J591 that showed saturation of PSMA expressed by the normal liver at 25 mg of huJ591 (Morris et al., 2005). A dose-dependent uptake in the liver with increasing mAb dose was observed, and optimal trade off was reached at a mAb dose of 25 mg. The unlabeled dose of huJ591 was administered intravenously within 5 min, immediately followed by injection of ^89^Zr-huJ591 within 1 min.

**Figure 3:**
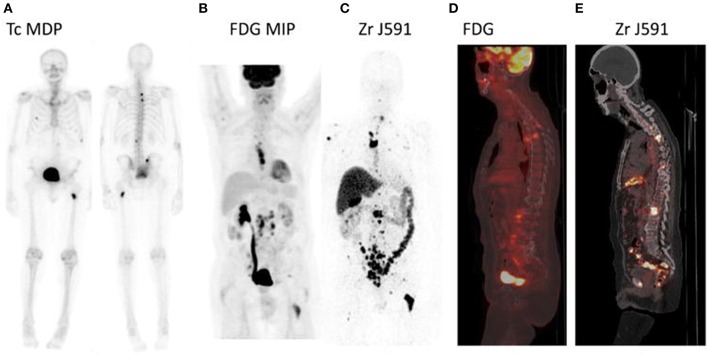
**^89^Zr-huJ591-PET and conventional imaging modalities of a patient with rising prostate specific antigen**. ^99m^Tc-MDP bone scan shows only a few lesions. FDG-PET shows nodal disease in the thorax, retroperitoneum, and pelvic region and a few bone lesions in the spine. Overall more bone lesions were seen on ^89^Zr-huJ591-PET than on FDG-PET, including multiple lesions in vertebrae, pelvic bones, ribs, and humerus. Targeting was also seen to the retroperitoneal and pelvic lymph nodes by ^89^Zr-huJ59-PET. **(A)** Anterior and posterior ^99m^Tc-MDP bone scan. **(B)** FDG-PET maximum intensity projection. **(C)**^89^Zr-huJ591 PET. **(D)** FDG-PET sagittal fused image. **(E)** ^89^Zr-hu J591 PET sagittal fused image. Reprinted with permission from Pandit-Taskar et al. (2014).

Pandit-Taskar et al. evaluated performance characteristics of ^89^Zr-DFO-huJ591 PET/CT for detecting metastases compared to conventional imaging modalities (baseline FDG-PET, ^99m^Tc-methylenediphosphonate (MDP) bone scans and CT scans) and pathology, to provide evidence for the use of ^89^Zr-huJ591 as an imaging biomarker. In a lesion-based analysis ^89^Zr-J591 imaging demonstrated superior visualization of bone lesions relative to conventional imaging, see Figure 3. However, detection of soft tissue lesions was suboptimal. A generalized lower tumor uptake was observed for soft tissue lesions compared to bone lesions. Low uptake was observed in normal bone and considered to be non-specific. Among the possibilities explaining a lower tumor uptake in soft tissue are: lower PSMA expression, absence of tumor in lesions presumed to be disease by CT and FDG-PET scan, or impaired accessibility of PSMA for intact mAbs. For the biopsy-confirmed lesions overall accuracy of ^89^Zr-J591 was 95.2% (20/21) for osseous lesions and 60% (15/25) for soft-tissue lesions. No data is provided on ^89^Zr-J591 uptake related to PSMA expression in tumor biopsies.

The authors conclude that ^89^Zr-huJ591 imaging is able to detect active disease earlier than conventional imaging, making PSMA an attractive target for diagnosis of prostate cancer. Despite the fact that just a small proportion of lesions were biopsied, statistical arguments indicated that ^89^Zr-huJ591 imaging detects 50% more bone lesions (occult disease) than bone scan. However, no single imaging modality can serve as gold standard, therefore a known site of disease was defined as any lesions identified by conventional imaging at baseline. Small lesions were most probably missed, while the treatment of patients after imaging was variable, which limits the detection of lesions through follow-up imaging.

The relatively long period post injection required for optimal imaging, may be a practical limitation of ^89^Zr-immuno-PET for routine application in diagnosis. Promising alternative ligands for molecular imaging of prostate cancer are smaller molecules as radiolabeled minibodies or urea-based small peptides, although none have been validated in controlled clinical trials for routine clinical use (Viola-Villegas et al., 2014). The perspectives of ^89^Zr-immuno-PET might be different when considering ^89^Zr-huJ591 for therapeutic approaches.

## ^89^Zr-labeled anti-MSLN in pancreatic and ovarian carcinoma

For several tumor types, such as pancreatic and ovarian carcinoma, no important drug targets are available. Exploitation of tumor-specific membrane proteins, even those without a known role in oncogenesis, as targets for delivery of potent drugs by ADCs is a promising approach. In this respect, a potential target molecule, with largely unknown biological function, is membrane-bound surface glycoprotein mesothelin (MSLN). It is minimally expressed by normal mesothelial cells, lining pleural, pericardial and peritoneal surfaces. Besides in mesothelioma, it is also highly overexpressed in 80-100% of pancreatic and ovarian cancers (and some other cancers). In preclinical studies with MSLN-expressing tumor bearing mice, ^89^Zr-anti-MSLN antibody MMOT0530A showed progressive and antigen-specific tumor uptake with micro-PET (Ter Weele et al., 2015).

Therefore, Lamberts et al. evaluated ^89^Zr-labeled MMOT0530A as a predictive imaging biomarker for treatment (in a phase I setting) with the ADC DMOT4039A, containing the MSLN-antibody MMOT0530A combined with the cytotoxic agent monomethyl auristatin E (Lamberts et al., 2016). For such an approach either the ADC itself can be labeled with ^89^Zr or the corresponding “naked” mAb, if available for human use. Labeling of the mAb part of the ADCs with ^89^Zr is well possible, but requires advanced analytical tools to prove that labeling is performed inertly. Assuming that both types of conjugates demonstrate similar biodistribution, which is a research question as such, PET imaging of the target will provide insight into drug distribution (tissue exposure, but also expression of the target and internalization of the antibody). Ideally, clinical ^89^Zr-immuno-PET studies with the “naked” mAb are performed before resources are put in the development of an ADC.

The aim of this imaging study was to assess biodistribution and tumor uptake, and the relationship between tumor uptake and MSLN expression, as well as response to treatment. Uptake in normal tissues was as expected, and did not indicate specific uptake, except for high hepatic uptake of ^89^Zr-MMOT0530A. This might be due to normal hepatic catabolism of the antibody, maybe slightly elevated by complex formation of the mAb with MSLN antigen shed into the circulation, as MSLN is not expressed on normal liver. Nevertheless, the uptake level in liver was similar to that of other antibodies such as trastuzumab and huJ591. Significant clinical toxicity, reported as dose limiting toxicity, were hypophosphatemia and hyperglycaemia and liver function abnormalities occurred in less than 10% of these patients. Tumor uptake of ^89^Zr-MMOT0530A was observed in 37 tumor lesions in 11 patients with pancreatic cancer and 4 patients with ovarian cancer, while 6 measurable tumor lesions visible on diagnostic CT in 4 patients were not detected by ^89^Zr-immuno-PET. Within patients a mean 2.4 ± 1.1-fold difference in uptake between tumor lesions was observed, indicating interlesional heterogeneity of tumor uptake.

Tumor uptake of ^89^Zr-MMOT0530A was correlated with MSLN expression levels determined with IHC scores (6 patients with pancreatic cancer and 4 patients with ovarian cancer). No correlation was found when the two tumor types were analyzed separately. Tumor uptake of ^89^Zr-MMOT0530A was not correlated with progression free survival, on both patient and lesion-based analysis.

The imaging dose for this study was considered to be sufficient, since the amount of tracer still present at 7 days p.i. was enough to clearly visualize the circulation. A first cohort of two patients received 37 MBq ^89^Zr-anti-MSLN (1 mg) and were imaged at days 2, 4 and 7 p.i. Patients in the second cohort (*n* = 10) received a total protein dose of 10 mg mAb, administered as a co-infusion. The biological half-life of ^89^Zr-anti-MSLN in cohort 1 was shorter than in cohort 2, most likely due to faster antibody clearance related to small amounts of shed MSLN antigen present in the circulation. Bioavailability of the imaging dose in the second cohort was considered sufficient to evaluate tumor uptake.

This was the first study aiming the use of ^89^Zr-immuno-PET as an imaging biomarker for whole body target expression and organs at risk for toxicity, to ultimately guide dosing, confirm delivery, and predict efficacy of the ADC. At this stage of development, however, ^89^Zr-immuno-PET was not able yet to add valuable information for individualized treatment decisions.

## ^89^Zr-labeled anti-CD20 mAbs in B cell lymphoma

Especially when using mAbs for radioimmunotherapy (RIT), ^89^Zr-immuno-PET may be applied to predict toxicity by assessment of biodistribution. This information may enable individualized treatment by optimizing dose schedules to limit unnecessary toxicity for patients.

RIT is used in the treatment of lymphoma, as this type of cancer is highly radiosensitive. More than 90% of B-cell non-Hodgkin lymphoma (NHL) express CD20, making it an attractive target for treatment. The transmembrane phosphoprotein CD20 is also expressed on mature B cells. The biological function of CD20 is still unclear. CD20 is highly expressed on the cell surface and is not rapidly internalized after antibody binding. The anti-CD20 antibody rituximab is widely used in both first-line, as well as subsequent treatment lines for patients with B-cell NHL. Anti-CD20 based RIT with yttrium-90 (^90^Y)-labeled-ibritumomab tiuxetan is currently approved for treatment of relapsed and refractory NHL (Mondello et al., 2016). Bone marrow toxicity of RIT is dose-limiting, and especially patients with bone marrow infiltration may suffer excessive hematotoxicity.

Rizvi et al. published a clinical study on ^89^Zr-immuno-PET to predict toxicity of RIT in NHL patients in order to guide individualized dose optimization (Rizvi et al., 2012). The aim of this study was to assess whether pre-therapy scout scans with ^89^Zr-ibritumomab tiuxetan can be used to predict biodistribution of ^90^Y-ibritumomab tiuxetan and the dose limiting organ during therapy. Patients received standard treatment of 250 mg/m^2^ rituximab 1 week before and on the same day prior to both ^90^Y-and/or ^89^Zr-ibritumomab tiuxetan (70 MBq) administrations. The correlation between predicted pre-therapy and therapy organ absorbed doses as based on ^89^Zr-ibritumomab tiuxetan images was high. Biodistribution of ^89^Zr-ibritumomab tiuxetan was not influenced by simultaneous therapy with ^90^Y-ibritumomab tiuxetan. Pre-therapy scout scans with ^89^Zr-ibritumomab tiuxetan can therefore be used to predict biodistribution and dose-limiting organ during therapy. These results indicate that ^89^Zr-immuno-PET may guide safe individualized therapy by optimizing the radioimmunotherapy dose of ^90^Y-ibritumomab tiuxetan.

The standard treatment with a high amount of cold rituximab before anti-CD20 based RIT, also administered to the patients in the study of Rizvi et al., is common practice to enhance the therapeutic index for RIT. It is thought that the usage of excess unlabeled mAb before RIT may reduce toxicity, in particular bone marrow toxicity. Preloading with unlabeled mAb might prevent normal tissue toxicity by providing a more predictable biodistribution of ^90^Y-labeled mAb, decreasing clearance rates, and prolonging its circulation half-life. This preload is assumed to clear the peripheral blood of circulating CD20-positive B cells and enhance tumor targeting of the ^90^Y-labeled antibody to tumor cells. However, supportive data for this approach is limited. It is unclear whether the preload may block subsequently administered ^90^Y-labeled anti-CD20 antibody, which might impair therapeutic effects.

Muylle et al. performed a study with ^89^Zr-rituximab-PET to explore the influence of a preload with unlabeled rituximab in five patients with CD20-positive B-cell lymphoma, scheduled for subsequent RIT with ^90^Y-labeled rituximab (Muylle et al., 2015). The aim of the study was to compare the distribution of ^89^Zr-rituximab without and with a preload of unlabeled rituximab (within the same patient) to assess the impact on tumor targeting and radiation dose of subsequent radioimmunotherapy with ^90^Y-labeled rituximab. PET scans were obtained at baseline after administration of 111 MBq ^89^Zr-labeled rituximab (10 mg). After 3 weeks, a standard preload of unlabeled rituximab (250 mg/m^2^) was administered, immediately followed by administration of 10 mg ^89^Zr-rituximab, and PET scans were acquired.

For the patients with B cell depletion (*n* = 3) tumor uptake without a preload was consistently higher. In patients with preserved circulating B cells (*n* = 2), 3 lesions showed less or no uptake without a preload, while other lesions showed higher uptake. The authors explain higher tumor uptake upon preload by improved biodistribution and prevention of sequestration of ^89^Zr-rituximab in the “antigen-sink,” consisting of CD20-positive B cells in the circulation and in the spleen. Impaired targeting of other tumor sites, however, is explained by partial saturation with unlabeled rituximab.

**Figure 4:**
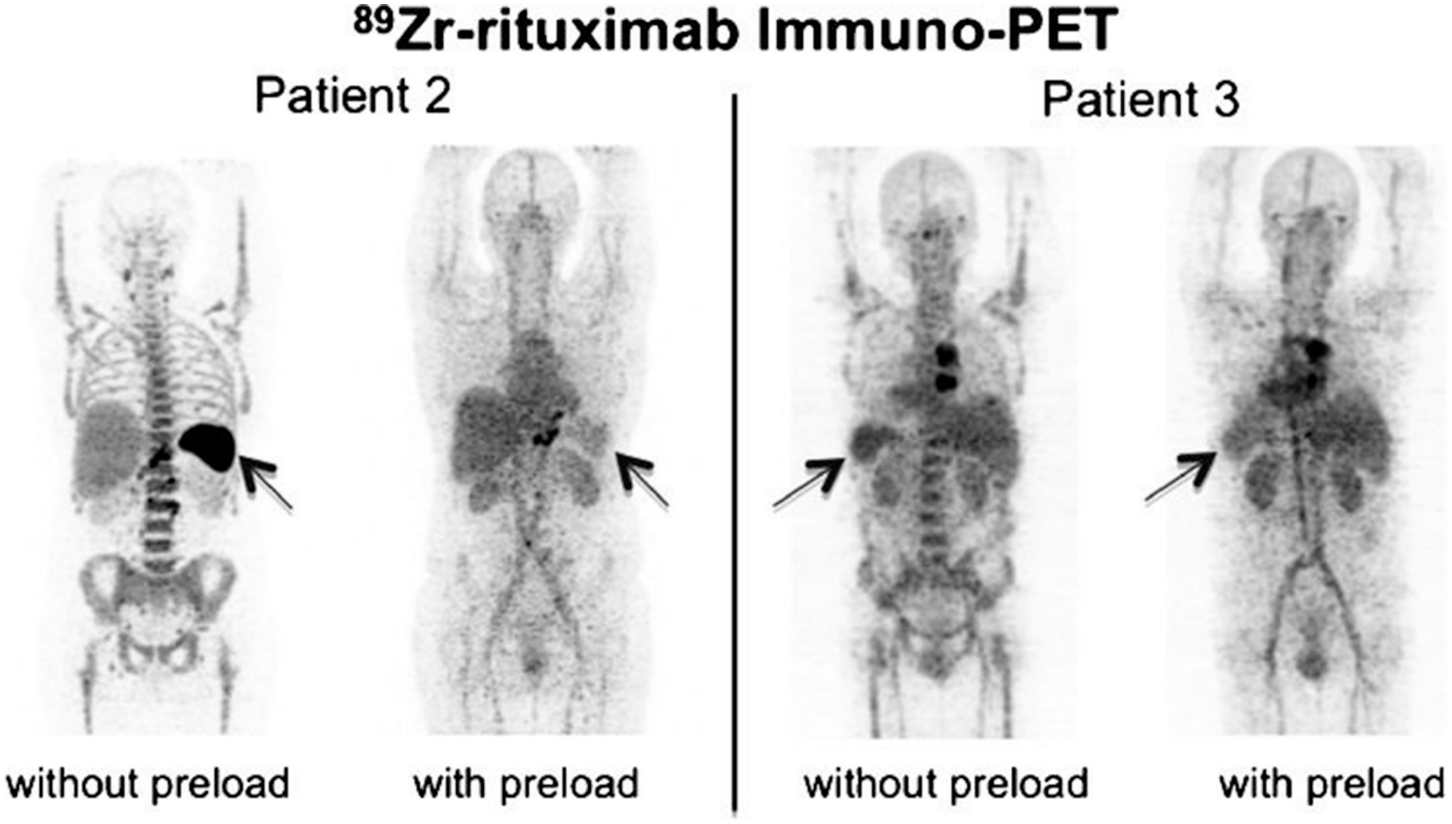
**^89^Zr-rituximab-PET images obtained 6 days after injection. (A)** Patient 2 without B cell depletion, anterior view. **(B)** Patient 3 with B cell depletion, posterior view. Reprinted with permission from Muylle et al. (2015).

For patients with preserved circulating CD20-positive B-cells (*n* = 2) without a preload of unlabeled rituximab, an increase in whole-body radiation dose of 59 and 87% was observed mainly due to increased uptake in the spleen, see Figure 4. The effective dose of ^89^Zr-rituximab was 0.50 milliSievert (mSv)/MBq without a preload and 0.41 mSv/MBq with a preload.

These results suggest that administration of the standard preload of unlabeled rituximab impairs tumor targeting of ^89^Zr-rituximab in patients with B-cell depletion, due to previous treatment with rituximab. These data suggest that common practice of preloading with unlabeled rituximab before RIT should be re-evaluated and reconsidered.

## Conclusions and future directions

In clinical trials with ^89^Zr-immuno-PET, two requirements should be met in order to realize its full potential. One requirement is that the biodistribution of the ^89^Zr-mAb (imaging dose) should reflect the biodistribution of the drug during treatment (therapeutic dose). An important pitfall to eliminate is a “false negative” ^89^Zr-immuno-PET due to absence of tumor uptake of the imaging dose, while there is tumor targeting of the therapeutic dose of unlabeled mAb. This situation may occur in case of expression of target antigen on normal tissue, or in case of a large tumor load with antigen expression. This causes an “antigen sink” that absorbs the tracer dose, leaving insufficient ^89^Zr-mAb to target all tumor lesions. Therefore, for each mAb, information should be obtained to assess whether a dose-dependent correlation between imaging dose and tumor uptake exists. Preferably a pilot study with different dose levels, within the same patient, is used to define the optimal ^89^Zr-mAb dose for imaging, i.e., the dose that reflects the therapeutic dose best. With respect to this, in case of co-administration of ^89^Zr-labeled and cold mAb, it should also be confirmed whether simultaneous administration is needed, or whether sequential administration immediate after each other is allowed.

Another requirement is that tumor uptake of the ^89^Zr-mAb reflects specific, antigen-mediated, tumor targeting. Next to specific uptake, also non-antigen mediated tumor uptake can occur, possibly caused by enhanced permeability and retention in tumor tissue. Even when tracer uptake is visualized in the tumor, no biological effect can be expected if tumor uptake is not primarily antigen-mediated (a “false positive” ^89^Zr-immuno-PET). Two clinical studies have reported a correlation between tumor uptake on PET and target expression in biopsies (Gaykema et al., 2013; Lamberts et al., 2016) indicating that tumor uptake on ^89^Zr-immuno-PET reflects specific, antigen-mediated binding. This would allow the use of ^89^Zr-immuno-PET as an imaging biomarker to assess target expression, as well as tumor targeting. However, for every mAb-antigen combination this has to be confirmed. In order to evaluate to which extent tumor uptake is driven by nonspecific and/or specific binding, studies correlating tumor uptake to target expression in biopsies, as well as exploration of kinetic modeling, may provide further insight.

Assuming these two crucial requirements are met, ^89^Zr-immuno-PET can be expected to predict toxicity and response to treatment.

For RIT, it was shown that biodistribution of ^89^Zr-rituximab can be used to predict biodistribution and the dose-limiting organ for subsequent treatment with ^90^Y-ibritumomab tiuxetan (Rizvi et al., 2012). This allows for future application of ^89^Zr-immuno-PET for individualized doze optimization of RIT with the aim to reduce the risk for toxicity. However, so far no clinical studies reported that ^89^Zr-immuno-PET predicted toxicity of mAb or mAb conjugate treatment.

Prediction of response to treatment for mAbs, is based on the assumption that the drug can only be effective if the target antigen is reached. Although, this is no guarantee for efficacy, as therapy failure may occur due to inadequate dosing of the mAb or intrinsic resistance mechanisms. On the other hand efficacy might occur at low antigen expression levels which is not visualized by ^89^Zr-immuno-PET. To improve prediction of efficacy of treatment, confirmation of tumor targeting of the drug can be combined with early response assessment. This combined approach may be able to predict whether adequate tumor targeting is followed by sufficient cytotoxicity. Clinical application of molecular imaging to guide individualized treatment may ideally consist of the following 3-step approach:

1. Tumor detection and staging of the disease with conventional imaging (e.g., FDG-PET/diagnostic CT)
2. Assessment of tumor targeting of the drug with molecular imaging (e.g., ^89^Zr-immuno-PET)
3. Early response assessment on treatment with conventional imaging (e.g., FDG-PET/diagnostic CT)

So far, the ZEPHIR study is the only study utilizing this 3-step approach, using an optimized ^89^Zr-mAb dose for imaging, and a visual classification for tumor uptake (Gebhart et al., 2016). This study reported that molecular imaging combined with early response assessment is able to predict response to treatment with T-DM1 in patients with HER2-positive breast cancer, opening avenues to cost-effectiveness studies and individualized treatment protocols. Still, this promising result can possibly benefit from improving criteria of positive uptake by quantitative analysis.

Currently, no standardized scale for visual scoring of ^89^Zr-immuno-PET is available, as opposed to scoring FDG-PET/CT scans by the Deauville criteria (Meignan et al., 2010). Imaging procedures, including data analysis and measurements of tumor uptake should be standardized and validated in order to use ^89^Zr-immuno-PET in clinical practice. As an example, problems with partial volume effects should be solved. Ideally, by linking imaging data with corresponding clinical outcome of a large set of patients, a criterion for positive tumor uptake can be defined.

Overall, these initial clinical trials have provided indications of the potential of ^89^Zr-immuno-PET as an imaging biomarker to assess target expression, as well as tumor targeting. The first results supporting application of molecular imaging for prediction of toxicity for RIT and response prediction for treatment with an ADC, form an important first step toward individualized treatment.

## Author contributions

YJ, GV performed the literature search and drafted the manuscript. CW, OH, NH, DV, JZ, and MH contributed significantly to the writing. All authors revised the work critically.

### Conflict of interest statement

The authors declare that the research was conducted in the absence of any commercial or financial relationships that could be construed as a potential conflict of interest.

## Abbreviations

ADC: antibody-drug conjugate
cmAb U36: chimeric monoclonal antibody U36
EGFR: epidermal growth factor receptor
FDG: 18F-fluoro-deoxy-glucose
HNSCC: head and neck squamous cell carcinoma
HER2: human epidermal growth factor receptor 2
HSP90: Heat shock protein 90
mAb: monoclonal antibody
MSLN: membrane-bound surface glycoprotein mesothelin
NET: neuroendocrine tumors
NHL: non-Hodgkin lymphoma
PET: positron emission tomography
p.i.: post injection
PSMA: prostate specific membrane antigen
RIT: radioimmunotherapy
T-DM1: trastuzumab-emtansine
TGF-β: Transforming growth factor-β
VEGF-A: vascular endothelial growth factor-A
^90^Y: yttrium-90
^89^Zr: zirconium-89.
